# Ambient Temperature-Responsive Mechanisms Coordinate Regulation of Flowering Time

**DOI:** 10.3390/ijms19103196

**Published:** 2018-10-16

**Authors:** Hendry Susila, Zeeshan Nasim, Ji Hoon Ahn

**Affiliations:** Department of Life Sciences, Korea University, Seoul 02841, Korea; susila_hendry@korea.ac.kr (H.S.); znasim09@korea.ac.kr (Z.N.)

**Keywords:** plant, ambient temperature, molecular mechanism, temperature perception

## Abstract

In plants, environmental conditions such as temperature affect survival, growth, and fitness, particularly during key stages such as seedling growth and reproduction. To survive and thrive in changing conditions, plants have evolved adaptive responses that tightly regulate developmental processes such as hypocotyl elongation and flowering time in response to environmental temperature changes. Increases in temperature, coupled with increasing fluctuations in local climate and weather, severely affect our agricultural systems; therefore, understanding the mechanisms by which plants perceive and respond to temperature is critical for agricultural sustainability. In this review, we summarize recent findings on the molecular mechanisms of ambient temperature perception as well as possible temperature sensing components in plants. Based on recent publications, we highlight several temperature response mechanisms, including the deposition and eviction of histone variants, DNA methylation, alternative splicing, protein degradation, and protein localization. We discuss roles of each proposed temperature-sensing mechanism that affects plant development, with an emphasis on flowering time. Studies of plant ambient temperature responses are advancing rapidly, and this review provides insights for future research aimed at understanding the mechanisms of temperature perception and responses in plants.

## 1. Introduction

Recent analyses indicate that global temperatures have steadily increased, by at least 0.6 °C in the last three decades and 0.8 °C in the last century [1]. Moreover, a recent simulation concluded that global temperature would continue to increase without any significant hiatus or slowdown [2]. Increments of global temperature followed by local climate change will likely have major effects on biodiversity and crop yields [3,4]. Plants respond to environmental changes by adjusting their growth and development to increase their survival and reproduction [5]; understanding the mechanisms behind these adjustments will provide essential information to help mitigate the effects of rising temperatures.

Environmental factors including photoperiod, temperature, and biotic-abiotic stresses affect flowering time [6]. Consistent with rising global temperatures, the flowering time of annual and perennial plants has changed over the past century [7,8]. For instance, warmer temperatures lead to early flowering of many crop plants, which shortens their development and eventually reduces yields [9,10]. Large-scale studies with natural accessions of the non-crop, model plant *Arabidopsis thaliana* showed temperature-dependent changes of flowering time [11,12].

Major ongoing efforts by laboratories around the world have identified several genes and possible mechanisms for ambient temperature-responsiveness [13,14,15]. Plant cells undergo several primary responses after a change in temperature, such as alterations of membrane fluidity and lipid composition, generation of secondary messengers, partitioning of import and antiport channels, changes of chromatin state, modulation of RNA processing or biogenesis, and protein translation and stability [16,17]. These primary responses, which may be important parts of the temperature sensing mechanism, are followed by secondary responses and eventually affect plant development [15]. Elucidation of the precise mechanism of temperature perception and identification of the primary molecular temperature sensors in plants require further investigation.

In this review, we focus on recent findings on the molecular mechanisms governing the primary and secondary ambient temperature responses in plants. The primary responses involve histone variants and modifications, DNA methylation, RNA processing, protein degradation through the 26S proteasome, and protein interaction/kinetics such as thermal reversion of phytochromes; the secondary responses involve the circadian clock and hormone signaling. This review starts with chromatin packing and DNA methylation, transcription, and RNA processing, then moves to protein-mediated mechanisms such as protein degradation, protein interactions and kinetics, and finishes with secondary responses, such as circadian clock and hormone signaling. We also discuss hypotheses that connect the molecular mechanism of ambient temperature sensing with plant developmental processes such as flowering time and hypocotyl elongation.

## 2. Histone Variants, Histone Modification, and DNA Methylation

Histones not only function to pack DNA into nucleosomes and chromatin; they also regulate the accessibility of DNA for replication, transcription, recombination, and repair [18]. The nucleosome is considered a stable protein–DNA complex, containing 146–147 base pairs of DNA wrapped around two copies of four histone proteins: Histone 2A (H2A), H2B, H3, and H4 [19]; however, the canonical histones undergo continuous turnover, especially the peripheral histones, H2A and H2B [20,21]. Histone variants, most of which are related to H2A, H3, and the histone linker H1 [22], can be substituted for the canonical histones in chromatin during different cellular processes and in different regions of chromatin, such as the centromere. These histone variants function in the regulation of transcription, DNA repair, and chromosome segregation [23]. Histone variants differ from their canonical histone counterparts by their amino acid sequences, physical properties, and the timing of their incorporation into chromatin during the cell cycle [24]. The canonical histones are synthetized and incorporated into the chromatin during the S phase of replication, but most of the histone variants are expressed and available throughout the cell cycle in a replication-independent manner [25]. In plants, histone variants are important during development and in the responses to environmental change.

One example of a histone variant that acts in the responses to environmental changes is H2A.Z, which is encoded by at least three genes (*HTA8*, *HTA9*, and *HTA11*) in *A. thaliana* and functions in thermosensing [26]. Several histone chaperones and ATP-dependent chromatin-remodeling complexes are responsible for H2A.Z deposition/depletion in nucleosomes [27,28]. The change of H2A-H2B dimers to H2A.Z-H2B dimers is regulated by the SWR1 complex, an ATP-dependent chromatin remodeling complex and a member of the SWI2/SNF family of ATPases [29]. In *A. thaliana*, at least 40 genes encoding SNF2 family proteins have been identified [30], although only PHOTOPERIOD INDEPENDENT EARLY FLOWERING 1 (PIE1) possesses an ATPase domain and is closely related to Swr1 from yeast [27,31,32]. Other homolog genes related to SWR1 complex, such as *ACTIN-RELATED PROTEIN 6* (*ARP6/SUF3*), *SWR COMPLEX 6* (*SWC6/SEF*), *ACTIN-RELATED PROTEIN 4* (*ARP4*), *YEAST AF9* (*YAF9*), and *SWR COMPLEX 4* (*SWC4*) have also been thoroughly characterized [32,33,34,35,36,37,38]. A forward genetic screen for mutants causing aberrant expression of *HEAT SHOCK PROTEIN 70* (*HSP70*) at low temperatures identified mutations in *ARP6*, which encodes a subunit of the SWR1 chromatin-remodeling complex, which regulates H2A.Z deposition; this indicates that H2A.Z prevents the expression of temperature-responsive genes, such as *HSPs*, at lower ambient temperatures [26]. Moreover, a recent genome-wide chromatin-immunoprecipitation sequencing (ChIP-seq) analysis found that at high ambient temperatures, H2A.Z levels (specifically HTA11) decrease in the +1 nucleosome, gene body, and the region surrounding the transcription start site (TSS) of warm temperature-responsive genes [39], suggesting that H2A.Z prevents the transcription of these genes at lower ambient temperatures.

Interestingly, the depletion of H2A.Z occurred only in warm temperature-responsive genes, not in all HTA11-bound genes. This suggested that other factors participate in the H2A.Z-to-H2A replacement in specific nucleosomes. Indeed, in the *hsf1abde* quadruple mutants, H2A.Z is not depleted from *HSP70* chromatin at warm temperatures, suggesting the importance of HEAT SHOCK FACTOR 1 (HSF1) in H2A.Z-to-H2A exchange [39]. The replacement of H2A.Z with H2A at warm temperature occurs rapidly, as H2A.Z occupancy at +1 nucleosomes and gene bodies is reduced 15 min after warm temperature (27 °C) treatment [39].

In addition to preventing inappropriate *HSP70* expression, H2A.Z also affects flowering time. For example, mutants of the loci encoding H2A.Z (*hta8 hta9 hta11* triple mutants) showed an early flowering phenotype [40]. Mutants of genes involved in H2A.Z deposition (for instance, *pie1*, *arp6*, *swc6*, *arp4*, and *yaf9*) also showed early flowering [32,36,37,41,42] similar to plants grown at warm temperatures. Moreover, these mutants are insensitive to temperature changes, indicating the exchange from H2A.Z to H2A is an early event in the perception of warm temperature, acting to adjust the length of the plant life cycle by accelerating the transition from the vegetative stage to the reproductive stage.

**Figure 1:**
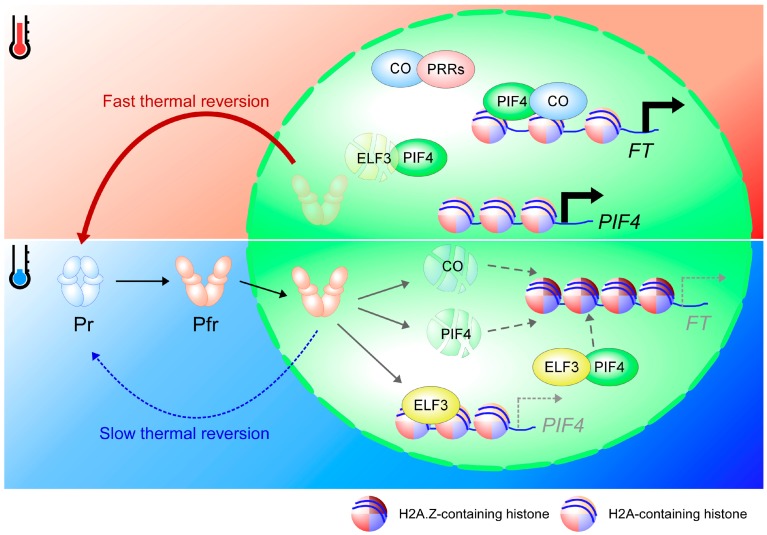
Mechanism of ambient temperature-responsive flowering involving histone variant eviction, phytochromes, and the circadian clock. At high ambient temperatures (red thermometer), H2A.Z is evicted and replaced by the canonical histone H2A. This facilitates the binding of PIF4 and CO to the *FT* promoter and activation of the transcription of *FT.* Fast thermal reversion of phyB at warm temperatures (bold red arrow) allows stabilization of CO and PIF4 to maintain robust *FT* expression. CO protein is stabilized by interacting with PRRs, which are circadian clock components, while the degradation of ELF3 by PIF4 alleviates the repression of *PIF4*. By contrast, at low ambient temperature (blue thermometer), H2A.Z is present in the *FT* promoter, blocking the access of transcription factors to the *FT* promoter. Slow thermal reversion (dashed blue arrow) of phyB at low temperatures induces the degradation of CO and PIF4 proteins, but maintains the stability of ELF3 to repress *PIF4*. ELF3 also likely blocks the function of PIF4 by direct interaction. Fragmented ovals indicate degraded proteins. Arrows and dashed arrows in the nucleus denote activation and inability to activate, respectively.

Approximately 300 genes involved in the flowering time pathway have been identified [6], but the H2A.Z and SWR1 complex only regulates a small subset of these flowering time genes, activating the expression of *FLOWERING LOCUS C* (*FLC*), *MADS AFFECTING FLOWERING 4* (*MAF4*), and *MAF5* [31,37,40,41,42,43]. FLC, MAF4, and MAF5 are closely related transcription factors involved in the repression of the key floral integrator genes *FLOWERING LOCUS T* (*FT*) and *SUPPRESSOR OF OVEREXPRESSION OF CONSTANS* (*SOC1*) [6]. However, the activation of *FLC*, *MAF4*, and *MAF5* by H2A.Z is opposite to the repressive effect of H2A.Z on *HSP* genes [26,31,39]. This effect may be due to the location of deposition of H2A.Z in the chromatin, as H2A.Z in gene bodies has a strong repressive effect, but H2A.Z in the +1 nucleosomes maintains the activity of some genes [44]. Nevertheless, the connection between the eviction of H2A.Z at warm temperatures and the acceleration of the phase transition is unlikely to be associated with repression of *FLC*, as *FLC* transcript levels are elevated at warm temperatures [45]. Furthermore, the flowering time of *flc*, *maf4*, and *maf5* mutants did not resemble that of plants grown at warm temperatures [46], indicating that another flowering time regulator is likely to be responsible for connecting the promotion of flowering time at warm temperatures and H2A.Z eviction. One of the simplest explanations may involve direct connection to the florigen gene *FT*, as H2A.Z is evicted from the TSS-proximal region of *FT* at warm temperatures [26], enabling transcription factors such as CONSTANS (CO) and PHYTOCHROME INTERACTING FACTOR 4 (PIF4) to access the *FT* promoter and thus enhance *FT* transcription [13,47,48] (Figure 1). Additionally, H2A.Z activates the expression of the microRNA loci *MIR156A*, *MIR156B*, and *MIR156C* [49,50]. Nevertheless, the possibility of miRNA156 functioning as a connector of warm temperature-mediated H2A.Z eviction and the acceleration of phase transition will need further investigation.

**Figure 2:**
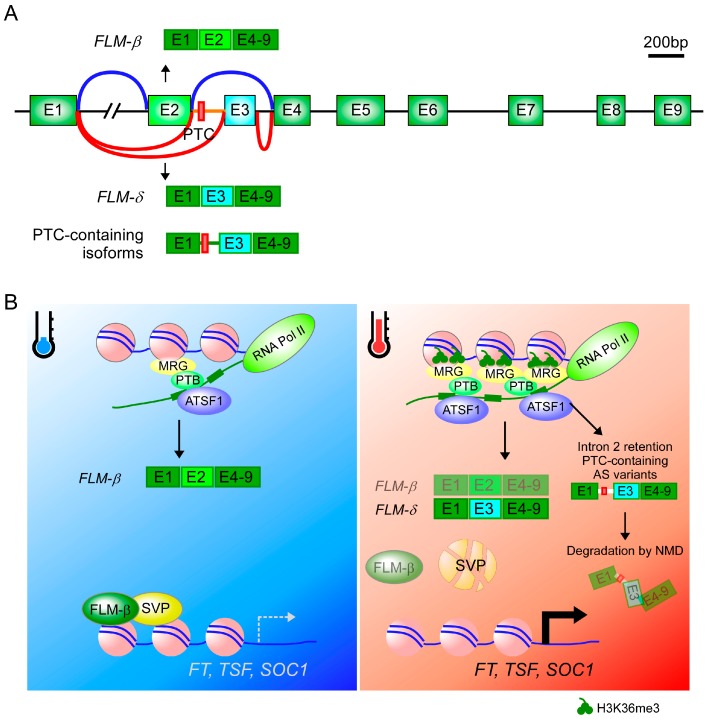
Possible regulation of alternative splicing of *FLM* at different ambient temperatures. (**A**) Schematic representation of the exon/intron structure of the *FLM* locus and possible alternative splicing at high ambient temperature (red lines) and low ambient temperature (blue lines). At low ambient temperature, the *FLM-β* transcript, which contains exon 2 (E2), is the predominant splicing variant, but at high-ambient temperature the *FLM-δ* transcript and premature termination codon (PTC)-containing isoforms are the predominant splicing variants. (**B**) H3K36me3 levels in the *FLM* locus are low at low ambient temperature (blue thermometer) allowing the robust production of *FLM-β*, the repressive form, to repress the expression of *FT*, *TSF*, and *SOC1* together with SVP protein. At warm temperature (red thermometer), the elevation of H3K36me3 levels at the *FLM* locus enhanced the production of other splicing variants such as the *FLM-δ* transcript and PTC-containing isoforms, which reduces the amount of *FLM-β*, probably through the tethering mechanism of splicing factor (SF1) and the Polypyrimidine Tract Binding Protein (PTB) to MRG, a histone reader. The PTC-containing isoforms are non-functional and degraded by nonsense-mediated mRNA decay (NMD). The lower level of *FLM-β* together with the degradation of SVP protein, which results in low abundant FLM-β-SVP repressor complex and thus release the repression of *FT* transcription. Faint oval and fragmented ovals indicate proteins with low abundance and degraded protein, respectively. Arrows and dashed arrows denote activation and inability to activate, respectively.

Although the exchange between histone variants and canonical histones is important for temperature responses in plants, other mechanisms such as chromatin modification may also play a role during acclimatization to warm temperatures [51,52]. For example, histone tails undergo various covalent modifications, such as methylation, that regulate binding by various transcription factors and chromatin proteins. The enrichment of histone H3 lysine 36 trimethylation (H3K36me3) at warm temperatures leads to differential splicing at the genome-wide level, probably through the H3K36me3-MORF RELATED GENE (MRG)-polypyrimidine tract-binding chromatin-adaptor mechanism [52,53] (Figure 2A,B). MRG proteins act as a histone reader that recognizes H3K4me3/H3K36me3 to modulate gene expression through the interaction with Histone H4-specific acetyltransferases, HISTONE ACETYLTRANSFERASE OF THE MYST FAMILY 1 (HAM1) and HAM2 [54,55]. Several flowering time genes are regulated by this process, including *FLOWERING LOCUS M* (*FLM*), *MAF2*, *PSEUDO-RESPONSE REGULATOR 3* (*PRR3*) and *PRR7*, which are differentially spliced in response to warm temperatures [52]. *FLM* and *MAF2* encode repressors of flowering [6,46]. Among the splice variants of *FLM*, the variant containing intron 2 has a premature termination codon (PTC) and is subject to non-sense mediated mRNA decay (NMD); therefore, this variant is non-functional and accelerates flowering time [56]. Moreover, like SWR1 complex mutants, the *set domain-containing protein 8* (*sgd8*) and *sgd26* mutants, which have a lesion in the SGD histone methyltransferase, are insensitive to temperature changes [26,52], and the mutants and over-expressing lines of the histone eraser *JUMONJI30* (*JMJ30*) have alterations of flowering time only at higher temperatures [45,52]. Therefore, H3K36me3 enrichment and H2A.Z depletion may occur as early events in the control of flowering time at high temperature.

The effect of temperature on DNA methylation has been also explored [57,58]. In the plant, DNA methylation occurs on cytosine residues in the contexts of CG, CHG, and CHH (H represents A, T, or C) with different methylation pathways [59,60]. CG methylation in gene bodies represents constitutively active loci, while methylation in the promoter region represents repression of the transcription of its associated gene(s) [61,62]. On the other hand, the non-CG methylation is critical to suppress the expression of transposable elements and small RNAs in the heterochromatic region [60,63]. The maintenance of CG methylation requires METHYLTRANSFERASE 1 (MET1) and CHG methylation requires CHROMOMETHYLASE 3 (CMT3) and CMT2, while CHH methylation requires DOMAINS REARRANGED METHYLASE 2 (DRM2) and CMT2, although it was recently shown that MET1 and CMT3 are also required for the maintenance of asymmetric CHH methylation at the CMT2 non-overlapping region [64,65].

A recent analysis of DNA methylation in Swedish *A. thaliana* accession revealed a strong correlation between CHH methylation and temperature. [57]. Higher CHH methylation levels were observed in plants grown at 16 °C, compared to 10 °C [57]. Although CHH methylation is regulated by two distinct pathways; firstly, via DRM2 in the canonical RNA-directed DNA methylation pathway and secondly via CMT2, which likely regulates the temperature-dependent CHH methylation levels [57,58]. Furthermore, a genome-wide association study approach also found a strong correlation between the CMT2 locus and temperature seasonality, supporting the hypothesis that the temperature-dependent CHH methylation response is likely to be regulated by CMT2 [66]. The accession containing the *CMT2_STOP_* allele displayed lower CHH methylation levels in the transposable element (TE)-bodies, resembling the *cmt2* mutants [66]. Indeed, *CMT2* is also involved in heat-stress tolerance, indicating a functional role of *CMT2* and CHH methylation in the temperature response [57,58,66].

Although the direct connection between temperature response, CHH methylation, and flowering time still remains elusive, it is known that *FLOWERING WAGENINGEN* (*FWA*) gene, probably one of the most studied DNA methylation targets, is involved in flowering time regulation [67]. The dominant *fwa-1* epiallele exhibits DNA hypomethylation within its promoter region, resulting in ectopic expression during the vegetative stage, which leads to late flowering [68,69]. However, the silencing of *FWA* in the vegetative tissue by DNA methylation suggests a minimal role of FWA in flowering time regulation [70]. Interestingly, PICKLE (PKL), a chromatin remodeling factor, plays a role in non-coding RNA-directed DNA methylation by maintaining the levels of CG, CHG, and CHH methylation [71]. *pkl-1* mutants exhibit late flowering and reduced the hypocotyl elongation at a warm temperature [72,73]. However, the lower ambient temperature-mimicking response of *pkl-1* mutants is likely through the function of PKL to repress the H3K27me3 repressive mark, which results in the activation of downstream target genes including *ELONGATED HYPOCOTYL5* (*HY5*) and *LEAFY* (*LFY*) [72,74]. Although the mechanism is still not clear, the presence of PKL appears to be crucial to repress H3K27me3 levels at warm temperatures [73]. Thus, it is tempting to speculate that the role of PKL in ambient temperature-responsive plant development also requires the regulation of DNA methylation, probably by elevation of CHH methylation levels at warm temperatures. Further experiment such as bisulfite sequencing in *pkl-1* mutants at different ambient temperatures might be necessary to verify this hypothesis.

## 3. Non-Coding RNAs

Two classes of non-coding RNAs, long non-coding RNAs (lncRNAs) and microRNAs (miRNAs), have been shown to play important roles in the regulation of flowering time [75,76,77,78]. We will briefly discuss these two classes:

### 3.1. Long Non-Coding RNAs (lncRNAs)

Long non-coding RNAs are long (>200 nucleotides) RNA transcripts without any apparent protein coding function [79]. Based on their origins, lncRNAs can be classified into five groups: stand-alone lncRNAs [also called large intergenic non-coding RNA (lincRNAs)], natural anti-sense transcripts (NATs), pseudogenes, long intronic non-coding RNAs, and a catch-all category that contains divergent transcripts, promoter-associated transcripts, and enhancer RNAs [80]. LncRNAs regulate transcription in *trans* or *cis* [81]. LncRNAs promote transcription directly by recruiting transcription factors or RNA polymerases, and indirectly by modifying the epigenetic marks on DNA and histones of the target loci [79,81]. Moreover, lncRNAs silence the expression of the target genes by direct interaction interfering elongation of RNA polymerase, RNA splicing, or polyadenylation [81,82].

In *Arabidopsis*, the *COOLAIR* and *COLDAIR* lncRNAs regulate flowering time with roles in vernalization-mediated repression of *FLC* [75,77,83]. *COOLAIR* is transcribed from the 3′ end of *FLC* in an antisense direction and *COOLAIR* transcripts can be classified into two groups based on their usage of two alternative polyadenylation sites: class I is proximally polyadenylated and class II is distally polyadenylated [76]. The autonomous pathway members FLOWERING CONTROL LOCUS A (FCA), FY, and FPA promote polyadenylation of *COOLAIR* transcripts at proximal sites [84,85]. Mutations in these genes result in distal polyadenylation in *COOLAIR* [85,86]. *COOLAIR* binds to the *FLC* locus and causes transcriptional repression of *FLC* during vernalization [77].

By contrast, *COLDAIR* is transcribed from the first intron of *FLC* in a sense direction upon cold treatment. Unlike *COOLAIR*, *COLDAIR* does not undergo polyadenylation [83]. In vitro transcription and pull-down assays along with RNA-immunoprecipitation assays showed that *COLDAIR* binds to the Polycomb repressive complex PRC2 in vivo. *FLC* transcript levels did not respond to vernalization in *COLDAIR* knockdown lines compared to wild-type plants and *FLC* expression was de-repressed in the knockdown lines upon shifting the plants to warm temperatures [83]. The vernalization-induced CURLY LEAF and H3K27me3 enrichment at the chromatin of the *FLC* locus was largely impaired in *COLDAIR* knockdown lines, revealing the importance of *COLDAIR* for recruiting PRC2 to the *FLC* chromatin [83]. Therefore, *COOLAIR* and *COLDAIR* have different modes of regulating *FLC* transcription.

Interestingly, a genome-wide expression profiling analysis identified at least 50 lncRNAs (11 NATs, two intronic lncRNAs, and 37 lincRNAs) that are differentially expressed at warm temperatures [87]. From these 50 lncRNAs, one lncRNA called *AtLnc428* or *FLOWERING LONG INTERGENIC NON CODING RNA* (*FLINC*) is involved in temperature-mediated flowering by repressing the transcription of *FT* [87]. However, further analyses are required to identify the exact targets of *FLINC* and the mechanism by which *FLINC* affects its targets. Nevertheless, as the expression of *FLINC* is downregulated at higher temperatures, it seems that *FLINC* modulates flowering time by repressing the positive regulators of flowering or by activating the expression of negative regulators. It will be of great interest to study the temperature-dependent flowering or thermomorphogenesis phenotypes of all 50 lncRNAs.

### 3.2. miRNAs

A recent study profiled the small RNA (sRNA) populations in four different tissues of *A. thaliana* grown at different ambient temperatures (15, 21, and 27 °C). About 0.6% of the total sRNA loci were found to be regulated in response to ambient temperature [88]. MicroRNAs (miRNAs), a subclass of endogenous sRNAs, are 21–22 nucleotides long with biologically important roles [89]. MiRNAs play a vital role in regulating a variety of plant developmental and physiological processes [90,91,92]. A number of miRNAs and their target genes have been shown to influence flowering time [93,94,95]. Microarray and northern blot experiments at two different temperatures identified 22 miRNA loci that are differentially expressed in response to ambient temperature changes [96]. Nine of these miRNAs (miR156a, g; miR159c; miR169a, b, d, h; miR319a, c) showed were upregulated at a lower ambient temperature (16 °C), and 13 miRNAs (miR163a; miR167a, d; miR171b; miR172a, c; miR395a; miR398a, b; miR399a, d, e; miR408a) were up-regulated at a higher ambient temperature (23 °C) [96].

In plants, miR156 is the major miRNAs governing the phase transition in development with the highest level of expression during the juvenile stage and gradually decreasing levels as the plant ages [95,97,98]. The targets of miR156 are *SQUAMOSA PROMOTER BINDING PROTEIN-LIKE* (*SPL*) genes, which regulate a number of genes involved in the floral transition and flower development including genes that promote flowering, such as *SOC1*, *AGAMOUS*-*LIKE 42* (*AGL42*), and *FRUITFULL* (*FUL*), and floral meristem identity genes, such as *APETALA1* (*AP1*) and *LFY* [99,100,101,102]. These genes, along with *FT*, promote the transition from the vegetative phase to the reproductive phase and subsequent floral development [100,103,104]. Overexpression of miR156 in *Arabidopsis* resulted in a prolonged vegetative phase and late flowering and mutants of miR156-targeted *SPL* genes exhibited a similar phenotype [105]. This repressive effect on phase transition of miR156 overexpression has been shown in other plant species including tomato (*Solanum lycopersicum*), rice (*Oryza sativa*), maize (*Zea mays*), red clover (*Trifolium pratense* L.) and switchgrass (*Panicum virgatum* L.), suggesting an evolutionally conserved function of miR156 in controlling the floral transition [91,98,106,107,108,109].

Additionally, miR172, which plays an opposite role to miR156, has been shown to participate in the ambient temperature-dependent regulation of flowering [96,110]. Levels of miR172 were higher in plants at 23 °C compared with 16 °C, while miR156 was regulated in an opposite manner [96]. Members of the *SPL* family promote flowering through the direct activation of miR172 [97,111]. Consistent with its opposite role to that of miR156, overexpression of miR172 resulted in early flowering by downregulation of a group of AP2-domain transcription factors including *TOE1*, *TOE2*, *TOE3*, *SCHLAFMÜTZE* (*SMZ*), *SCHNARCHZAPFEN* (*SNZ*) and *AP2*, which play an important role in the floral transition [90,112,113,114,115]. Overexpression of miR172 in rice [116] and maize [117] also produced a similar early flowering phenotype.

In addition, levels of mature miR172 and pri-miR172a were reported to be anti-correlated with SHORT VEGETATIVE PHASE (SVP) protein activity, implying direct repression of MIR172 by SVP [96,118]. SVP and FLM bind directly to the *MIR172a* promoter [119,120], supporting the possibility that these transcription factors might act as upstream repressors of *MIR172a*. These results suggest that at low ambient temperatures the high levels of the SVP–FLM-β complex (see Section 4 and Section 5) also repress *MIR172a* expression and hence delays flowering.

Although the level of miR156 is high at lower ambient temperature, pri-miR156a,c, and d are present at higher levels at higher temperatures, indicating that miR156 is regulated by temperature at the processing level [121]. Unlike animal miRNAs, the precursors of plant miRNAs show high variability in their structure and fold-back length, varying from 50 to 900 nucleotides [122,123]. Although many components involved in plant miRNA biogenesis have been uncovered, the processing mechanism of each miRNA family seems to be slightly different depending on the structure of the precursor [123,124]. There are at least two major processing mechanisms found in plant miRNAs (loop-to-base and base-to-loop processing), where the upper stem, lower stem, and bulges are the structural determinants that are important for the accuracy and efficiency of miRNA hairpin processing [121,123,124,125,126,127]. Indeed, mutations in the lower stem, upper stem, and bulges of pri-miR156a affect the production of miR156, thus altering the flowering time [121,128,129]. Interestingly, although the majority of the mutations in the upper stem and lower stem reduce the efficiency of miR156 formation at both lower and higher ambient temperatures, the effect of the disruption of base pairing in upper stem 2 (S2) was extraordinarily severe at lower temperature, suggesting the importance of S2 in the regulation of temperature-mediated flowering time [121,128,129]. Therefore, the S2 region likely acts as a structural determinant for temperature-responsive miR156 hairpin processing [121]. Although the exact mechanism is still unclear, temperature changes probably affect miR156 processing by fine-tuning the base-pair stability and opening dynamics in the hairpin structure [128,129].

Additionally, miR399 along with its target *PHOSPHATE2* (*PHO2*) regulates ambient temperature-mediated flowering in a FCA-dependent manner [78,130]. miR399 levels were higher at 23 °C when compared with 16 °C [78,96]. Overexpression of miR399 and mutation of its target gene *PHO2* caused early flowering at 23 °C, probably because of high *TWIN SISTER OF FT* (*TSF*) expression, but no effect on flowering was seen at a low ambient temperature (16 °C) [78]. However, the mechanism explaining how miR399 mediates temperature changes awaits further work.

## 4. Alternative Splicing

In plants, MADS-box transcription factors constitute a large gene family and play vital roles in almost all developmental processes [131,132,133,134]. Some MADS-box transcription factors act as key upstream regulators of flowering time, including FLC and other FLC clade members [i.e., FLM (=MAF1), MAF2, MAF3, MAF4, and MAF5] [135,136]. All these *FLC* clade genes along with *SVP* have been implicated in the thermosensory pathway [46,137,138]. Loss of function of *SVP* results in almost complete insensitivity to ambient temperature changes, whereas the loss of *FLM* results in partial temperature insensitivity with an early flowering phenotype [131]. *SVP* has been reported to act mainly in the thermosensory pathway downstream of the autonomous pathway members FCA and FVE [138]. Additionally, genetic variations of *SVP* and *FLM* govern the variation in flowering time among natural accessions of *A. thaliana* [139,140,141]. Nevertheless, although the role of MADS-box proteins in ambient temperature-responsive flowering is well known, how temperature affects the transcription and splicing of MADS box genes has only been understood recently.

*FLM* undergoes alternative splicing [142]. The alternative splicing events in *FLM* result in a large number of splice variants, among which *FLM-β* and *FLM-δ* are the main forms. Both splice variants differ in the incorporation of either the second or third cassette exon and both transcripts produce protein [120,138]. *FLM-β* is the predominant splice isoform at a low ambient temperature (16 °C), while *FLM-δ* is abundant at a high ambient temperature (27 °C) [120,131]. Strikingly, overexpression of *FLM-β* and *FLM-δ* under the control of the CaMV 35S promoter resulted in opposite phenotypes: *FLM-β* overexpression delayed flowering, but *FLM-δ* overexpression accelerated flowering [120]. To further validate the role of both splice variants, a recent study employed CRISPR/Cas9 to specifically delete the *FLM-β* and *FLM-δ* isoform-specific exons, generating lines that only expressed either *FLM-β* or *FLM-δ* transcripts [143]. Lines that produced the repressive *FLM-β* variant without *FLM-δ* flowered late, whereas lines that produced *FLM-δ* without *FLM-β* showed early flowering, further confirming the repressive role of *FLM-β* over a range of ambient temperatures. However, the flowering phenotype of *FLM-δ*-producing lines was comparable to *flm* knockout mutants, suggesting the minimal role of *FLM-δ* isoform in the regulation of flowering time [143].

Recent work identified ARABIDOPSIS SF1 HOMOLOG (AtSF1), which regulates *FLM* splicing, plays an important role in the recognition of the 3’ splice site of *FLM* by directly binding to the intron branch point [144]. AtSF1 protein has an RNA recognition motif (RRM) domain [144] and a mutation in the RRM domain affects the production of alternatively spliced *FLM-β* transcripts. However, the loss of AtSF1’s RRM domain affects the alternative splicing of only a specific subsets of transcripts, suggesting the possibility of other yet-to-be-discovered factors that function in the alternative splicing of *FLM* transcripts [144].

Interestingly, Sureshkumar and colleagues showed that high ambient temperatures downregulate *FLM* expression by alternative splicing coupled with nonsense-mediated mRNA decay (AS-NMD) [56]. They identified at least 39 thermosensitive splice variants from the *FLM* locus at high ambient temperature (27 °C) [56,143]. Most of them contain a PTC in intron 2 and they are cleared out by the NMD pathway [145], which seems to be important to regulate overall *FLM* expression [56] (Figure 2A,B). *FLM* expression levels were significantly higher in the NMD-impaired *upf* mutants compared to wild-type plants, suggesting that NMD is indeed involved in the post-transcriptional repression of *FLM*. However, the exact molecular mechanisms underlying how alternative splicing senses elevated ambient temperatures and how the splicing machinery conveys the “message” by reducing fidelity of splice site recognition and/or usage to make faulty transcripts are yet to be elucidated.

Circular RNAs (circRNAs) are produced by circularization of exonic sequences as a product of alternative splicing [146]. CircRNAs have recently been shown to play important roles in plant growth and development [147,148]. For example, a recent study showed that *FLM-β* circRNA has a temperature-dependent expression pattern like *FLM-β* mRNA, suggesting a possible role of this circRNA to ensure high abundance of FLM-β proteins at low temperatures [147]. However, further experiments are needed to show the biological significance of *FLM-β* circRNA. Similarly, circRNAs were detected from loci of other MADS-box transcription factors including *FLC*, *SEPALLATA3* (*SEP3*), and *SEEDSTICK* (*STK*) [147] but no information is available related to their temperature-dependent expression patterns or biological significance of their circRNAs.

*MAF2*, another gene in the *FLC* clade, also undergoes temperature-responsive alternative splicing in response to temperature and causes floral repression at low temperatures [149,150]. Like FLM, MAF2 is a major determinant of natural variation of flowering time in *A.* *thaliana* [151]. Low ambient temperatures induce expression of *MAF2var1*, the predominant *MAF2* splice variant, which encodes a protein that forms a repressive complex with SVP to inhibit flowering. High ambient temperatures induce expression of the intron-retaining splice variant *MAF2var2*, which contains a PTC [149,150,152]. The truncated protein produced from *MAF2var2* lacks part of the K-domain and the entire C-domain, which are important for homo- and hetero-dimer formation of MADS-box transcription factors [153,154]. Therefore, the protein produced from *MAF2var2* cannot interact with SVP to form a repressive complex. 

Another important splice variant is *MAF2var5*, in which a PTC is introduced by skipping of the sixth exon [152]. Although expression levels of *MAF2var5* were low and its expression was somewhat insensitive to ambient temperature changes, *MAF2var5* protein was able to interact with SVP [152]. Interestingly, overexpression of *MAF2var5* resulted in early flowering, implying that *MAF2var5* competes with FLM for the interaction with SVP, possibly in a similar way as FLM-δ protein does [120]. These results suggest an additional MAF2–SVP module for sensing lower ambient temperatures and repressing flowering in parallel with FLM-SVP [152]. Taken together, these results highlight the significance of ambient temperature-driven alternative splicing in the regulation of flowering time in *Arabidopsis*.

## 5. 26S Proteasome-Dependent Protein Degradation 

**Figure 3:**
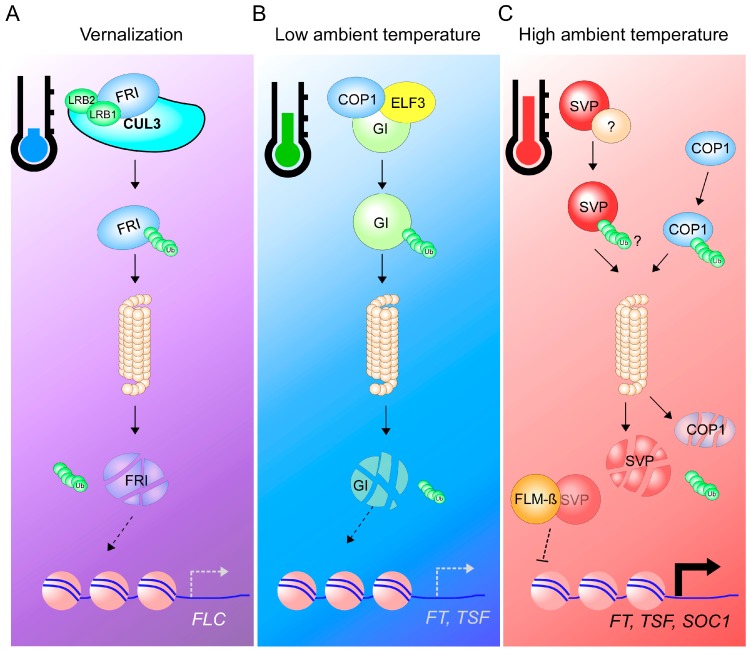
Temperature-dependent degradation of flowering time regulator proteins by the 26S proteasome. (**A**) Degradation of FRIGIDA (FRI) protein by the 26S proteasome. LRB1 and LRB2, members of the BTB protein family, along with CUL3A ubiquitin-E3 ligase polyubiquitinate FRI proteins under cold treatment (vernalization). The poly-Ub-tagged FRI proteins are then degraded by the 26S proteasome pathway, resulting in insufficient quantities of FRI proteins, which fail to repress *FLC* expression. This contributes to the vernalization-dependent repression of *FLC* in winter annuals, thereby accelerating flowering. (**B**) Low ambient temperature-induced degradation of GIGANTEA (GI) by the 26S proteasome. At low ambient temperatures, COP1, an ubiquitin-E3 ligase, along with ELF3 regulates levels of GI protein, which is an activator of flowering, through the 26S proteasome pathway. As a result, *FT* expression is reduced and flowering is delayed. (**C**) High ambient temperatures result in the degradation of SVP protein. Unknown ubiquitin ligase enzymes ubiquitinate SVP. This leads to reduction in the levels of the FLM-β-SVP repressive protein complex, thereby de-repressing the expression of floral activator genes (*FT*, *TSF*, and *SOC1*). Also, at high ambient temperatures, COP1 protein is unstable. These complex temperature-responsive systems ensure plants’ successful reproduction under fluctuating temperature conditions. Faint oval and fragmented ovals indicate proteins with low abundance and degraded protein, respectively. Arrows and dashed arrows denote activation and inability to activate, respectively. A dashed T-bar indicates a failure of suppression of transcription of target genes.

FRIGIDA (FRI), another flowering time regulator that is important in the vernalization pathway, is degraded via the 26S proteasome system [155]. *FRI* encodes a coiled-coil protein that induces *FLC* expression to repress the expression of the flowering pathway integrators *FT*, *SOC1*, and *LEAFY* [156,157,158]. High levels of FLC result in late flowering of the winter *Arabidopsis* accessions. These natural accessions begin their life cycles during the fall and undergo vegetative growth, then flower during the next spring [159]. By contrast, the summer annual *Arabidopsis* accessions have low *FLC* expression levels due to the absence of functional FRI and hence flower early; they complete their life cycles in a single growing season. LIGHT-RESPONSE BTB1 (LRB1) and LRB, two BTB (Bric-a-Brac/Tramtrack/Broad Complex) proteins, along with the CULLIN3A (CUL3A) ubiquitin-E3 ligase directly interact with FRI in vitro and in vivo, leading to proteasomal degradation of FRI during prolonged cold treatment (Figure 3A). This temperature-dependent degradation of FRI leads to the reduction of *FLC* expression and hence causes early flowering [155].

In addition to the CULLIN3A-BTB E3 ligase system, the RING-finger E3 ligase CONSTITUTIVE PHOTOMORPHOGENIC1 (COP1) was also identified as a regulator of ambient temperature-responsive flowering. COP1 regulates light-dependent processes such as photomorphogenesis, circadian oscillation, and floral transition [160,161]. The *cop1* mutants showed a temperature-insensitive early flowering phenotype at 16 °C and 23 °C in an *FT*-dependent manner. COP1 protein stability is regulated by ambient temperature with more COP1 protein accumulation at a low ambient temperature (16 °C) than at higher temperature (23 °C) [162]. COP1, along with EARLY FLOWERING 3 (ELF3), regulates the protein stability of GIGANTEA (GI) [161], a central player in the photoperiod and circadian clock pathways that directly activates *FT* expression [163]. High COP1 protein levels resulted in more GI degradation at low ambient temperatures, whereas GI proteins were stable even at low temperatures in *cop1* mutants, confirming the ambient temperature-dependent degradation of GI by COP1. Treatment with the 26S proteasome inhibitor MG132 reduced GI protein levels at low temperature, confirming the mechanism underlying the temperature-dependent ubiquitination of GI [162] (Figure 3B). Thus, the COP1–GI module seems to participate in the regulation of the floral transition at low ambient temperature [162]. 

Furthermore, COP1 along with DE-ETIOLATED 1 (DET1) are also important for hypocotyl growth at a warm-temperature [164]. COP1 conveys warm temperature signal mediated by HY5 protein degradation in the nucleus and stabilizes PIF4 protein [164,165]. Therefore, COP1 is likely to be regulated by ambient temperature in several ways; for instance, by protein stabilization at a lower ambient temperature and by nuclear transport at a higher ambient temperature [162,165]. Further experiments are required to determine whether these two mechanisms are interdependent. 

On the other hand, *SVP* and *FLM* interact genetically [120,142] to repress flowering at a range of ambient temperatures [120,131,142]. Also, FLM proteins produced from both *FLM-β* and *FLM-δ* transcripts are co-expressed with SVP and interact physically with SVP to form two different protein complexes [120,131]. These findings suggest that particular *FLM* splice variants govern the activity of the resulting SVP–FLM complex. Further supporting this hypothesis, SVP and FLM-β share a number of transcriptional targets [119,120]. However, in contrast to *FLM*, *SVP* transcript levels were not strongly affected by ambient temperature [131,138]. Rather, ambient temperature regulates SVP protein levels post-translationally, as SVP protein is rapidly degraded by the 26S proteasome pathway in response to warm ambient temperatures, thereby affecting the abundance of the repressive SVP–FLM-β complex [131] (Figure 3C). Therefore, post-translational protein regulation constitutes an important layer of floral regulation, acting by fine-tuning protein quantities of several major floral regulators. However, regulation at the post-translational level needs further study to answer important questions such as what is the E3 ubiquitin ligase that ubiquitinates SVP protein?

## 6. Thermal Reversion of Phytochromes

The light irradiation from the sun positively correlates with the environmental temperature [166], causing the diurnal temperature to change between day and night [167,168]. Therefore, the effect of light on plant development (photomorphogenesis) is closely linked with the effect of the temperature (thermomorphogenesis) [169]. For example, both light and temperature share a common pathway to regulate hypocotyl elongation through the DET1–COP1–HY5 pathway [170] as well as through the PHYB–PIF4 pathway [171]. Moreover, phytochromes regulate seed maturation/germination and flowering time in light intensity/photoperiod- and temperature-dependent manners, suggesting the central role of phytochrome is to regulate not only light-dependent plant development but also temperature-dependent plant development [172,173,174,175,176].

Recent publications from the Casal group [177] and the Wigge group [171] shed light on the function of phytochrome B (phyB) as a thermostat in *Arabidopsis*. The conversion of Pr and Pfr states of phytochromes are not only affected by the light but also by the temperature [178]. As the conversion from inactive Pr state to active Pfr state is induced by light, the Pfr state can also be spontaneously converted into the Pr state in a light-independent reaction called thermal reversion [179]. Thermal reversion involves the conversion of the Pfr:Pfr homodimer (D2) to a Pfr:Pr heterodimer (D1), which is considered a slow step, and followed by the conversion to Pr:Pr (D0), which is two times faster than the first reaction [177,180]. The conversion from Pfr into Pr is accelerated at warmer temperatures, as the temperature affects the conversion of D2 into D1 in the presence of light [177], and D1 into D0 in the dark [171]. Therefore, phyB is proposed to have a dual function as a photoreceptor and a thermosensor.

There are five phytochromes in *Arabidopsis*: phyA, phyB, phyC, phyD, and phyE [181,182]. Mutation of the phytochromes (in *phyABDE* or *phyABCDE* mutants) makes the plant unable to sense environmental temperature changes, so that the plant shows constitutive warm temperature responses, such as elongated hypocotyls and early flowering time [171,175,183]. Therefore, considering that thermal reversion is slow at lower temperature, the active form Pfr will have more time to exert its function at lower temperature, repressing developmental processes such as hypocotyl growth and flowering time. Indeed, the association of phyB with the promoters of its target genes is stronger at low temperature than at higher temperature [171]. Furthermore, the interaction of phyB with PIF6, which inhibits thermal reversion in vitro, is in agreement with the hypothesis that phyB and PIFs function as co-repressors [178,184]. The phyB–PIF3 complex undergoes mutual degradation to attenuate the light signaling process [185].

Phytochromes control flowering time by affecting several important flowering time regulators. For example, phyB facilitates CO protein degradation in the morning, but phyA and cryptochrome stabilize CO protein in the afternoon [186]. Another example is the transcription factor PIF4, as phyB facilitates the degradation of PIF4 [187]. An additional target is the Tandem Zinc-finger-Plus3 (TZP) transcription factor, a positive regulator of *FT* expression [188]. However, unlike CO and PIF4, phyB does not affect the stability of TZP; rather, phyB recruits TZP into nuclear photobodies to access the *FT* chromatin [188], suggesting a dual role of phyB as a repressor and activator of flowering time. Indeed, both *phyB* mutants and *phyB-*overexpressing lines show early flowering phenotypes by regulating the expression of *FT* at different times [189]. Therefore, phyB acts as a multi-purpose protein in day/night conditions to control the expression of *FT*. As both CO and PIF4 are required for the induction of *FT* transcription [47], the slow thermal reversion of Pfr might facilitate CO and PIF4 protein degradation at lower ambient temperature, resulting in late flowering (Figure 1). Nevertheless, more experiments are required to study the effect of thermal reversion of phytochromes in the regulation of flowering time, especially in the regulation of *FT* transcription, as thermal reversion require both spatial and temporal fine-tuning.

The mechanism of temperature-dependent thermal reversion is not exclusive to phytochromes. The thermal reversion of phototropin blue light photoreceptors in *Marchantia polymorpha* (Mp*PHOT*) is accelerated at warmer temperatures [190], indicating temperature-dependent thermal reversion might be a common mechanism for adaptation to fluctuating temperature conditions. Although the role of phototropins in plant development is likely to be minor, phototropin regulates several biological processes such as chloroplast movement, stomatal opening, and CO_2_ assimilation rate [190,191,192]. Interestingly, cryptochrome 2 (CRY2), the blue light photoreceptor, regulates flowering time in a temperature-dependent manner by activating *FT* expression together with CRYPTOCHROME-INTERACTING BASIC-HELIX-LOOP-HELIX1 [174,193]. Moreover, CRY1 regulates thermomorphogenesis by interacting with PIF4 [194]. Therefore, whether cryptochromes could act as a thermostat like phytochromes and phototropins is an interesting question.

## 7. Circadian Clock Entrainment and Compensation as an Output of Temperature Perception

The circadian clock is an internal time-control mechanism with an approximately 24-h rhythm period. The circadian clock consists of three common components (i.e., inputs, circadian oscillator, and outputs) [195]. Inputs are daily external cues (for instance, light and temperature) adjusting the internal circadian oscillator to maintain the 24-h rhythm and eventually affect a broad range of biological processes (output) including plant growth, development, and adaptation [196,197]. The circadian clock is regulated by a series of feedback loops interlocking transcriptionally and translationally, which are composed of a set of genes that can be divided into four major groups, morning-phased components [*CIRCADIAN CLOCK ASSOCIATED 1* (*CCA1*) and *LATE ELONGATED HYPOCOTYL (LHY*)], day-phased components [*PRR9*, *PRR7*, *NIGHT LIGHT-INDUCIBLE AND CLOCK-REGULATED GENE 1* (*LNK1*), and *LNK2*], afternoon-phased components [*REVEILLE 8*], and evening-phased components [*PRR5*, *TIMING OF CAB EXPRESSION 1* (*TOC1*), *CCA1 HIKING EXPEDITION*, *LUX ARRHYTHMO* (*LUX*), *BROTHER OF LUX ARRHYTHMO*, *ELF3*, and *ELF4*] [198].

Ambient light and temperature are two main inputs that control the circadian clock. For example, prolonged darkness causes the expression of core clock genes to become arrhythmic, and light intensity increases the period length [199]. Temperature shortens the period, i.e., the clock oscillates faster at warm temperatures [200,201]. Temperature has two main effects on the circadian clock. They are temperature compensation, a mechanism allowing the clock to maintain a relatively constant period [202], and temperature entrainment, a mechanism allowing the clock to reset as an adaptation to environmental changes [203]. 

Several studies with circadian clock mutants showed that *PRR7*, *PRR9*, *CCA1*, and *LHY* function in temperature compensation [204,205]. However, a recent study showed that temperature feeds into the circadian clock through the evening complex of ELF3, ELF4, and LUX [206]. Mutations in *ELF3*, *ELF4*, and *LUX* induce constitutive expression of warm temperature-induced genes, indicating that warm temperature represses evening complex function [206,207]. Chromatin-immunoprecipitation analysis showed that the binding of ELF3 to ELF4 decreases at warm temperatures, thus reducing the repression of target genes by the evening complex [208], although the mechanism is still not clear. FLOWERING BASIC HELIX-LOOP-HELIX 1 regulates the expression of *CCA1* and affects the period length at a warm temperature [209]. In addition, cold temperature affects the alternative splicing of *CCA1* and regulates plant acclimatization to cold temperatures [210]. Other circadian clock component genes, such as *LHY*, *PRR3*, *PRR5*, *PRR7*, *PRR9*, and *TOC1*, are also alternatively spliced in response to the temperature change, partly through the function of the RNA/DNA binding protein SICKLE [211,212]. Therefore, the regulation of circadian clock components by temperature can occur at multiple levels, for instance, at the splicing or post-translational levels [195].

The circadian clock acts as an internal cue in the regulation of flowering time. Both circadian clock and photoperiod tightly regulate the expression and protein stability of key flowering time regulators, such as *GI*, *CO*, *PIF4*, *PIF5*, and *FT* [195,213,214]. CO, PIF4, and GI directly bind to the *FT* chromatin to activate the transcription of *FT*. The expression of *CO* is repressed by the CYCLING DOF FACTOR (CDF) transcription factor, which is activated by CCA1 and LHY in the morning and is repressed by PRR9, PRR7, and PRR5 in the afternoon [215,216]. In the afternoon, CDF protein is degraded by GI, which makes a complex with FKF1 [217], allowing the accumulation of *CO* transcripts at dusk. As *CCA1α*, the main spliced form, is highly expressed in cold temperatures, further activation of *CDF* will repress the expression of *CO* and delay flowering time [210]. Meanwhile, day-phased and evening-phased components such as PRR9, PRR7, PRR5, and TOC1 stabilize CO protein during the day by direct interaction [218]. The expression levels of *PRR9*, *PRR7*, and *TOC1* increase at warm temperatures [206], which probably further stabilizes CO and accelerates flowering time (Figure 1).

The evening complex, ELF3-ELF4-LUX, directly represses the expression of *PIF4* and *PIF5* [219]. At warm temperatures, the binding of the evening complex to chromatin is reduced and eventually promotes the expression of *PIF4* and *PIF5* to accelerate flowering [208,220]. ELF3 directly interacts with PIF4 in the evening complex to reduce the binding of PIF4 to its target genes, which attenuates PIF4 activity [221]. ELF3 can make a complex with COP1 to destabilize GI protein and reduce the expression of *CO* and *FT* [161]. Furthermore, the floral repressor SVP is stabilized by direct interaction with ELF3, which likely allows further repression of *FT* transcription by SVP [222]. SVP protein is degraded at warm temperatures by the 26S proteasome pathway [131], which is coincident with the reduction of ELF3 activity at that temperature. A recent biochemical analysis showed that ELF3 protein is stabilized by phyB [221]. As the thermal reversion of phytochromes can serve as a temperature sensor in the plant, the interaction of phyB with several clock components, including CCA1, LHY, TOC1, GI, ELF3, ELF4, and LUX, probably has an important function in temperature entrainment and compensation [223,224,225]. There is a significant overlap in the binding of ELF3, LUX, and phyB proteins to the chromatin of their target genes [220]. Furthermore, *phyB* and the evening complex genes regulate a similar subset of genes and affect the same developmental processes, such as hypocotyl growth and flowering time [171,220,226]. Rapid thermal reversion of Pfr of phyB at warm temperatures likely abolishes the interaction between the evening complex and phyB, thereby lowering the suppression of target genes and allowing ELF3 degradation through PIFs [221] (Figure 1). Therefore, the alteration of the circadian clock probably acts as a secondary process to maintain the robustness of temperature-regulated developmental processes in plants. This alteration of the clock might fine-tune the developmental processes under fluctuating temperature conditions and temperature-dependent alternative splicing of circadian clock component genes might play a crucial role in regulating plant development.

## 8. Plant Hormones as Secondary Signals in Temperature Perception

Plant hormones relay signals from endogenous and exogenous cues, conferring developmental flexibility to flowering [227]. Gibberellic acids (GAs) are the best studied hormones in flowering, brassinosteroids (BRs), abscisic acid (ABA), jasmonic acid (JA), salicylic acid (SA), cytokinin (CK), and ethylene (ET) play a role in regulating flowering [228,229]. We will discuss the roles of GA, BR, and JA in ambient temperature-responsive flowering.

### 8.1. GAs, Key Flowering Time Regulators

The GA signaling pathway constitutes one of the four major flowering pathways and is tightly linked to ambient temperature-mediated flowering [230]. The GA-deficient *ga1-3* mutants strongly suppressed flowering in response to elevated temperature under short day (SD) conditions [11,231]; however, they were only moderately late flowering under long day (LD) conditions [11]. These results suggest a requirement for GAs under non-inductive photoperiodic conditions.

GA signaling is mediated by a class of nuclear-localized proteins, known as DELLAs [232]. The *Arabidopsis* genome contains five *DELLA* genes [233] and all these DELLA proteins are regulated post-translationally by different levels of GAs, which triggers degradation of DELLA proteins via the ubiquitin-proteasome system [234]. DELLA proteins physically interact with a number of transcriptional regulators. Such interactions interfere with the DNA-binding capacity of these transcription factors [235]. PIF4, one of the target transcription factors of DELLAs, plays an important role in the activation of *FT* expression at warm ambient temperature [47,48]. After interacting with DELLA proteins, PIF4 cannot bind to DNA [236,237]. These results suggest that GAs affect thermosensing by modulating DELLA–PIF4 interactions and the interaction with PIF-like transcription factors [238]. 

**Figure 4:**
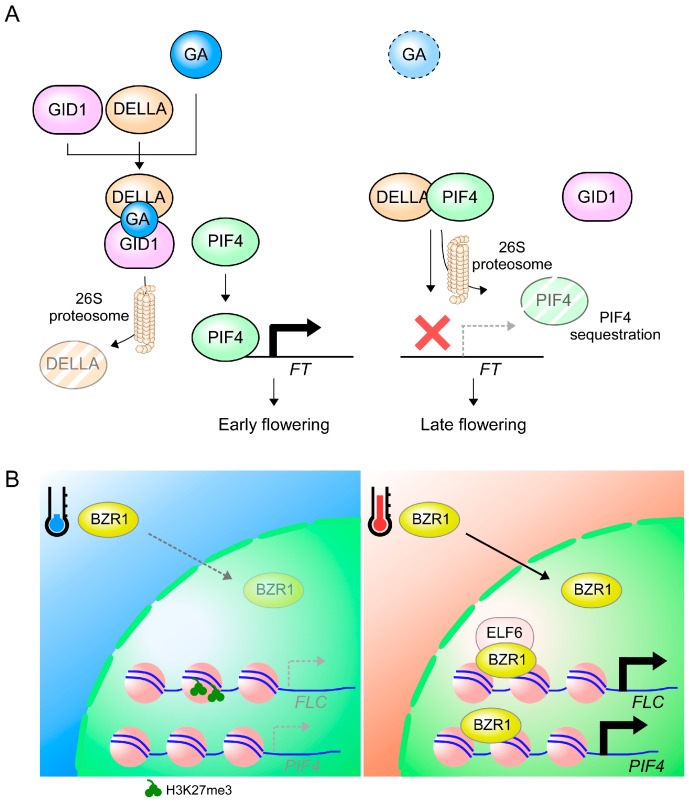
Hormone-regulated floral regulation. (**A**) Role of GAs in the regulation of flowering. In the presence of GA (left panel), GA induces the binding of GID1 with DELLA proteins, which initiates the ubiquitination and subsequent degradation of DELLA proteins. As a result of DELLA degradation, PIF4 can bind to the *FT* locus and induce *FT* expression, which leads to early flowering. However, in the absence of GAs, DELLA proteins induce the 26S proteasome-mediated degradation of PIF4 (right panel). As a result, PIF4 cannot bind to the *FT* locus and *FT* expression cannot be induced, resulting in delayed flowering. Arrows and dashed arrows denote activation and inability to activate, respectively. (**B**) The cellular localization of BZR1 is regulated by ambient temperature. At low ambient temperatures (blue thermometer), BZR1 is mainly localized in the cytoplasm, whereas at warm temperatures (red thermometer), BZR1 is localized in the nucleus and it interacts with ELF6 and allows rapid activation of *FLC* through the removal of H3K27me3. BZR1 also binds to the *PIF4* promoter to activate its transcription. Faint oval and fragmented ovals indicate proteins with low abundance and degraded protein, respectively.

Moreover, DELLAs can affect transcriptional events via mechanisms other than sequestration [235]. For example, DELLAs can trigger degradation of their bound proteins [239]. Although the degradation mechanism does not apply to all DELLA-bound targets (for instance, CO) [240], they do mediate degradation of PIFs via the ubiquitin-proteasome system [239] (Figure 4A). These findings suggest that GAs indirectly modulate temperature sensing and plant responses through sequestration and degradation of PIF transcription factors.

The importance of GA in temperature-dependent flowering is also seen in Kalanchoe plants. In some species of Kalanchoe that do not flower naturally, exogenous application of GA_3_ induced flowering. Interestingly, this induction of flowering was found to be temperature-dependent. At lower temperature (25/20 °C for day/night), up to 67% of the GA_3_-treated Kalanchoe plants flowered. However, at high temperature (30/25 °C for day/night), the Kalanchoe plants did not flower regardless of the concentration of the applied GA_3_ [241]. Although the molecular mechanism by which temperature modulates the effect of exogenous GA_3_ application is yet to be elucidated, higher temperature seems to negate the GA_3_-induced early flowering in *Kalanchoe* plants.

### 8.2. BRs

BRs, a group of steroid hormones, are widely distributed in plants and regulate a number of growth and developmental processes as well as responses to different stresses [242,243]. BRASSINAZOLE-RESISTANT 1 (BZR1) is a prominent transcription factor in thermomorphogenesis, highlighting the important role of BR in temperature responses [244,245]. Based on forward genetic screening, several BR biosynthesis genes were identified as responsible for hypocotyl elongation under warm temperature conditions [244]. Reverse genetics screen coupled with genome-wide expression analyses also identified BR biosynthesis genes as responsible for root elongation under warm temperature conditions [245]. Therefore, it seems that BR is responsible for increases in the length of the cells in response to warm temperature [244,245,246]. Warm temperature promotes nuclear localization of the transcription factor BZR1 via a phosphorylation-dependent mechanism, although the mechanism is still unclear, as BZR1 phosphorylation was not altered at low or high temperature [244,247]. In the nucleus, BZR1 activates the expression of *PIF4* and interacts with PIF4 to promote thermomorphogenesis [244,248].

In the regulation of flowering time, several experiments reported different roles of BR as a floral repressor or an activator [249,250]. The BR receptor mutant *brassinosteroid insensitive 1* (*bri1*) in the Wassilewskija (Ws) background showed a late flowering phenotype, indicating the floral promoting function of BR [250]. The opposite phenotype was observed in the Colombia (Col-0) background, where the *bri1* mutation accelerated flowering time [249]. In the Ws ecotype, the *bri1* mutation further delayed the late flowering phenotype of the autonomous pathway mutant *luminidependens-3* (*ld-3*) [250]. The *bri1* mutation increased the expression of *FLC* (around 1.4-fold) in the *ld-3* mutant background, indicating the role of BR in flowering probably occurs mainly through *FLC* regulation [250]. However, the subtle increase in *FLC* expression levels in *ld-3 bri1-201* mutants seems insufficient to explain the strong late flowering phenotype of *ld-3 bri1-201* mutants [250], indicating that BR probably also regulates flowering time through mechanisms other than *FLC* regulation, for instance, through the activation of *PIF4*. 

At least in the Col-0 ecotype, BR acts as a flowering repressor, as it induces the expression of the floral repressor *FLC* via two different mechanisms [249,251]. First, the transcription factor BZR1 transcriptionally represses *FLD*, a negative regulator of *FLC*; therefore, it promotes *FLC* expression [251]. Moreover, BZR1 interacts with EARLY FLOWERING 6 (ELF6), a chromatin modifier that possess H3K27me2/3 demethylation activity [249,252]. BZR1 likely recruits ELF6 to remove the repressive H3K27me3 marks at the *FLC* locus and promotes *FLC* expression [249]. Moreover, expression analyses showed the activation of other *FLC* clade members such as *FLM*, *MAF4*, and *MAF5* upon BR treatment [249]. Therefore, it seems that BR and BZR1 have a dual function in flowering time regulation at warm temperatures: (1) promotion of flowering by induction of the expression of *PIF4* and (2) attenuation of flowering by induction of *FLC* expression (Figure 4B). 

### 8.3. Is JA Connected to Ambient Temperature-Mediated Flowering?

JA, a fatty acid-derived compound, is associated with plant defense responses against pathogens [253]. However, JA has also been proposed to participate in flowering time regulation in several plant species [254,255]. The JASMONATE-ZIM domain (JAZ) family of transcriptional repressors, which are essential for JA signaling, are targeted by the F-box protein CORONATINE-INSENSITIVE PROTEIN 1 (COI1) for degradation [256,257]. JA induces the interaction between these two proteins. JAZ proteins repress the activity of transcription factors, including the bHLH-containing MYC2 protein, that coordinate JA responses. Hence, by the degradation of JAZ proteins, JA induces the de-repression of JA responses. The *coi1* mutants showed early flowering under both LD and SD conditions, suggesting that the COI1-dependent JA signaling pathway represses flowering in *Arabidopsis* [255,258].

JA might modulate the access of TARGET OF EARLY ACTIVATION TAGGED 1 (TOE1) and TOE2 proteins to the *FT* promoter, via degradation of JAZ family transcriptional repressors [258]. JA treatment enhanced the binding of TOE1 and TOE2 transcription factors to the *FT* locus in a COI1-dependent manner, leading to reduced *FT* expression and hence delayed flowering [258]. In addition, a recent study showed reduced expression of miR156 and higher expression of miR172, two important ambient temperature-responsive miRNAs, in early flowering JA-deficient *precocious* (*pre*) rice mutants, in which oxide synthase, a key enzyme in JA biosynthesis, is impaired [259], although the molecular mechanism is not well-understood. As these results suggest potential crosstalk among JA signaling, the aging pathway, and ambient temperature signaling, further investigation is required to clarify the relationship between ambient temperature and JA signaling.

## 9. Conclusions and Future Perspectives

After sensing warm or cold temperature, plants readjust their developmental processes such as flowering time and hypocotyl/root elongation to adapt to the new environmental conditions. In this review, we summarize the recent findings on several components that may act as thermostats and thermosensing mechanisms, mostly in the model plant *A. thaliana*. We discuss several prominent primary potential thermosensors or thermosensing mechanisms such as histone variant deposition and eviction, histone modification, mRNA splicing, protein degradation and cellular localization, lncRNA and miRNA production, and thermal reversion of phytochromes. These primary mechanisms affect secondary processes such as transcriptional regulation, hormone signaling, and circadian clock function, and eventually shape the development of the plant.

Despite recent progress made to uncover the ambient temperature-sensing mechanism, several important questions remain and we discussed possible future analyses of ambient temperature responses in plants. For example, whether the current model could be applied in natural environmental conditions remains an important question. Instead of using constant ambient temperature, further experiments need to be done under natural conditions or at least under fluctuating ambient temperature conditions mimicking field temperatures. These strategies could evaluate whether the primary temperature-sensing responses are still applicable in short-term or long-term temperature changes. Furthermore, connecting plant developmental processes with other temperature-associated responses such as changes in membrane fluidity, enzyme kinetics, and molecular structure would improve our understanding regarding ambient temperature-responsive development in *Arabidopsis* and in crop plants.
